# Bioprosthetic mitral valve thrombosis less than one year after replacement and an ablative MAZE procedure: a case report

**DOI:** 10.1186/1749-8090-5-18

**Published:** 2010-03-29

**Authors:** Omar Saeed, Byron R Williams, Melvin Ku, Omar M Lattouf

**Affiliations:** 1 Department of Medicine, Emory University, Atlanta, Ga, USA; 2 Division of Cardiology, Emory University, Atlanta, Ga, USA; 3 Department of Medicine, Michigan State University, East Lansing, MI, USA; 4 Division of Cardiothoracic Surgery, Emory University, Atlanta, Ga, USA

## Abstract

Occurrence of bioprosthetic valve thrombosis less than a year after replacement is very uncommon. Here, we describe a case of a 57 year old male, who presented 10 months after receiving a bioprosthetic mitral valve replacement with a two week history of dyspnea on exertion, worsening orthopnea and decreased exercise tolerance. Echocardiography revealed severe mitral regurgitation (MR), thrombosis of the posterior mitral leaflet, left atrial (LA) mural thrombus and a depressed left ventricular ejection fraction of twenty-five percent. Given severe clot burden and decompensated heart failure (New York Heart Association - NYHA class III) repeat sternotomy was done to replace the bioprosthetic mitral valve and remove LA mural thrombus. MR was resolved postoperatively. This brief report further reviews promoting factors, established guidelines and management strategies of bioprosthetic valve thrombosis.

## Background

Bioprosthetic mitral valves are advantageous over mechanical valves as their incidence of thrombosis, pannus formation and embolic events are significantly lower. This disparity in thromboembolic events as compared to mechanical valves avoids a need for chronic anticoagulation in many patients receiving bioprosthetic valve replacement [1]. However, bioprosthetic mitral valves can present with thrombosis shortly after replacement in high risk patients not maintained on anticoagulation, leading to severe valve incompetence and cardiac decompensation, as noted in the following case.

## Case Presentation

A 57 year old male with a past medical history of chronic atrial fibrillation, a depressed ejection fraction of 25%, and severe MR underwent mitral valve replacement with a bioprosthetic Mosaic valve (Medtronic Inc., Minneapolis MN) and a complete left and right sided MAZE procedure. Post operative transthoracic echocardiogram (TTE) showed a competent mitral valve tissue prosthesis and the patient was discharged on warfarin. Anticoagulation was discontinued three months after valve replacement and the patient remained in sinus rhythm on electrocardiography.

**Figure 1:**
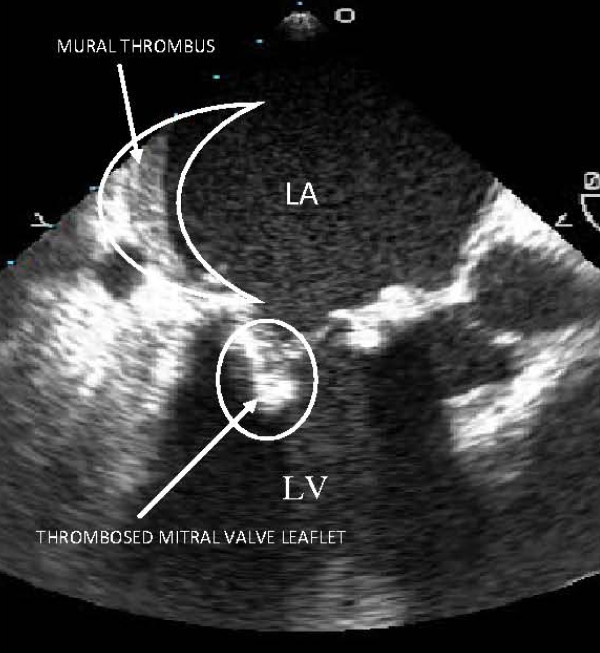
**TEE showing mural thrombus in the left atrium and a thrombosed bioprosthetic mitral valve**.

Ten months following mitral valve replacement, the patient presented with a two week history of progressive dyspnea on exertion, orthopnea and weight gain. TTE revealed severe mitral valve stenosis and mitral regurgitation, with a mean gradient of 8.5 mmhg (max. gradient -- 25.5 mmhg) across the mitral valve, and restricted motion of mitral leaflets. On transesophageal echocardiogram (TEE), a mitral mass was observed on the posterior leaflet along with mural thrombus in a dilated left atrium measuring 4.90 cm in diameter (figure 1). Ejection fraction was 25%. The patient was started on intervenous diuretics and anticoagulation with Heparin, however due to severity of clot burden and progressive decompensated heart failure (NYHA class III) repeat sternotomy was performed.

Intraoperatively, mural clot was debrided from the free and posterior left atrial walls. The mitral bioprosthesis was incompetent with pannus growing on the posterior leaflet. This valve was excised and replaced with a 25 mm On-X mechanical valve (On-X Life Technologies, Inc., Austin TX.) that functioned without leakage after placement. Postoperatively, the patient had improved exercise tolerance and was discharged on indefinite warfarin therapy.

## Discussion

Clinically significant bioprosthetic valve thrombosis is considered uncommon, however, its incidence on routine echocardiographic surveillance is reported as high as six percent [2]. Specific risk factors that promote formation of bioprosthetic valve thrombosis include large LA size, atrial fibrillation leading to decreased transvalvular flow, decreased LVEF, prior history of thromboembolic events and a hypercoagulable diathesis [3-5]. In the present case, two risk factors were present including an enlarged LA measuring 4.90 cm in diameter and a decreased EF of 25%. Moreover, radioablative MAZE lines are areas of damaged LA endothelium which can serve as a nidus for thrombus formation. Due to the presence of several pro-thrombotic risk factors prolonged anticoagulation with warfarin could have assisted in prevention of thrombus formation and valve dysfunction.

Although freedom from long term anticoagulation is a feature of bioprosthetic valve replacement, this case calls for a brief review of current anticoagulation guidelines following bioprosthetic valve replacement and a radioablative MAZE procedure. The 2008 American Heart Association (AHA) and American College of Cardiology (ACC) guidelines state that high risk patients with any of the following risk factors including atrial fibrillation, LV dysfunction with EF < 30%, recurrent thromboembolic events or a hypercoagulable condition meet a class I indication for indefinite anticoagulation [6]. To reduce the risk of thrombus formation after a radio ablative procedure for atrial fibrillation the 2007 Heart Rhythm Society (HRS) expert consensus statement recommends a minimum two month period of warfarin anticoagulation that should be continued indefinitely if the patient's CHADS_2 _score is greater or equal to 2 [7]. These guidelines further emphasize a beneficial role of prolonged anticoagulation in high risk patients.

This case also calls for frequent post operative echocardiographic monitoring in non- anticoagulated patients with risk factors promoting valve thrombosis. The 2007 European Society of Cardiology (ESC) guidelines [8] recommend initial follow up 6-12 weeks post operatively with TTE imaging. Thereafter, yearly clinical assessment is prudent and repeat TTE imaging is dependent on development of new cardiac symptoms. TEE can be considered if TTE is of poor quality, in cases of suspected prosthetic valve dysfunction or endocarditis. However, this case illustrates that high risk patients not on prolonged anticoagulation can develop significant thrombus and valve dysfunction prior to becoming symptomatic. More frequent clinical cardiac assessment and/or TTE imaging in high risk asymptomatic patients might allow earlier detection of bioprosthetic valve dysfunction by clot formation.

According to the 2007 ESC guidelines [8], once valve thrombosis reaches clinically significant obstruction, management between anticoagulation and surgery rests on the level of critical illness. Surgery is performed in critically ill patients unless it is not immediately available or in patients unlikely to survive surgery due to significant co-morbidities, where fibrinolysis is considered as an alternative. In non-critically ill patients, if their baseline degree of anticoagulation was inadequate then heparin and aspirin are initiated and thrombus is reassessed by echocardiography. If there is no resolution of valve thrombosis then surgery versus fibrinolysis is considered. In cases of persistent or recurrent valve thrombosis despite adequate anticoagulation surgical replacement is performed.

Management of non-obstructive prosthetic valve thrombosis depends on the occurrence of thromboembolism (TE) and thrombus size [8]. Initially, anticoagulation is optimized. In patients with evidence of TE, surgery is performed immediately if thrombus size is >10 mm, otherwise it is delayed. In patients without TE, surgery is performed for persistent thrombus of >10 mm on adequate anticoagulation and fibrinolysis is considered in high risk patients with multiple co morbidities.

In the present case, obstructive valve thrombosis, large clot burden and critical illness made surgical replacement a reasonable and successful treatment option.

## Conclusion

Bioprosthetic mitral valve thrombosis is an under recognized complication in high risk patients that leads to rapid valve incompetence. Post-operatively, patients must be stratified in high or low risk categories, and anticoagulation should be maintained indefinitely for high risk patients. If anticoagulation is not maintained for individualized reasons then a semi-annual TTE within the first year and annually thereafter may detect subclinical bioprosthetic valve thrombosis in asymptomatic patients.

## Consent

Written informed consent was obtained from the patient for publication of this case report and accompanying images. A copy of the written consent is available for review by the Editor-n-Chief of this journal.

## Competing interests

OML discloses that he has served as a consultant and received research grants from Medtronic Corporation and On-X Life Technologies. The rest of the authors declare that they do not have any competing interests.

## Authors' contributions

OS: Resident physician, provided pre-operative care and primary author.

BRW: Cardiologist, provided pre-operative care and advice during the manuscript writing process.

MK: Assisted in writing and preparing manuscript.

OL: Cardiothoracic Surgeon, performed the bioprosthetic valve repair and provided advice during the manuscript writing process.

All authors have read and approve the final manuscript.
